# miR-194 Inhibits Innate Antiviral Immunity by Targeting FGF2 in Influenza H1N1 Virus Infection

**DOI:** 10.3389/fmicb.2017.02187

**Published:** 2017-11-07

**Authors:** Keyu Wang, Chengcai Lai, Hongjing Gu, Lingna Zhao, Min Xia, Penghui Yang, Xiliang Wang

**Affiliations:** ^1^State Key Laboratory of Pathogen and Biosecurity, Beijing Institute of Microbiology and Epidemiology, Beijing, China; ^2^Beijing 302 Hospital, Beijing, China

**Keywords:** FGF2, H1N1, lung injury, miR-194, RIG-I signaling pathway

## Abstract

Fibroblast growth factor 2 (FGF2 or basic FGF) regulates a wide range of cell biological functions including proliferation, angiogenesis, migration, differentiation, and injury repair. However, the roles of FGF2 and the underlying mechanisms of action in influenza A virus (IAV)-induced lung injury remain largely unexplored. In this study, we report that microRNA-194-5p (miR-194) expression is significantly decreased in A549 alveolar epithelial cells (AECs) following infection with IAV/Beijing/501/2009 (BJ501). We found that miR-194 can directly target FGF2, a novel antiviral regulator, to suppress FGF2 expression at the mRNA and protein levels. Overexpression of miR-194 facilitated IAV replication by negatively regulating type I interferon (IFN) production, whereas reintroduction of FGF2 abrogated the miR-194-induced effects on IAV replication. Conversely, inhibition of miR-194 alleviated IAV-induced lung injury by promoting type I IFN antiviral activities *in vivo*. Importantly, FGF2 activated the retinoic acid-inducible gene I signaling pathway, whereas miR-194 suppressed the phosphorylation of tank binding kinase 1 and IFN regulatory factor 3. Our findings suggest that the miR-194-FGF2 axis plays a vital role in IAV-induced lung injury, and miR-194 antagonism might be a potential therapeutic target during IAV infection.

## Introduction

Influenza A virus (IAV) infection is the leading cause of pneumonia-related deaths worldwide (Herold et al., 2015). Most IAV infections result in respiratory illness, with severe cases resulting in acute lung injury (ALI) (Cao et al., 2009; Jain et al., 2009; Short et al., 2014). The innate immune response provides the first line of defense against viruses and other pathogens. IAV infection exposes the host cell to single-stranded genomic RNA and double-stranded RNA intermediates of viral replication, which are recognized by endosomal toll-like receptor (TLR) 3 or TLR7 (Rehwinkel et al., 2010) and the cytoplasmic RNA helicase retinoic acid inducible gene I (RIG-I) (Pichlmair et al., 2006); such recognition drives the activation of antiviral responses during viral infection. Type I interferon (IFN) plays a critical role in the antiviral immune response mainly by inducing cellular resistance to viral infection and apoptosis of virus-infected cells. The RNA helicase RIG-I is a major innate immune sensor, which functions by binding and reacting to 5’ double-stranded RNA. Binding of viral RNA to the RIG-I helicase domains triggers its interaction with mitochondria-associated antiviral signaling protein (MAVS), which leads to the production of proinflammatory cytokines downstream of activation nuclear factor kappa-light-chain-enhancer of activated B cells beta, and the production of type I IFNs and IFN-stimulated genes via nuclear translocation of IFN regulatory transcription factor 3 (IRF3) (Durbin et al., 2013; Iwasaki and Pillai, 2014; Liu et al., 2015; Tavares et al., 2017). However, the mechanisms underlying the regulation of this pathway require further characterization (Weber-Gerlach and Weber, 2016).

MicroRNAs (miRNAs) are a class of abundant, highly conserved small non-coding RNAs (18–25 nucleotides [nt]). They suppress gene expression by binding to the 3′-untranslational region (UTR) of target mRNAs, thereby providing a novel mechanism of post-transcriptional regulation. miRNAs are gaining recognition as having central roles in diverse biological processes including immune responses, inflammation, and tumorigenesis. Host cellular miRNAs are involved in the regulation of IAV replication *in vitro* (Loveday et al., 2012; Wolf et al., 2016; Rosenberger et al., 2017), and miRNAs that target host mRNAs to modulate antiviral responses following influenza infection have been identified (Globinska et al., 2014). miRNA-194-5p (miR-194), a typical p53-responsive miRNA, plays a reversal role in proliferation, migration, and the invasion of various human cancers such as prostate cancer, gastric cancer, glioma cancer, and non-small-cell lung carcinoma. It has been described as a suppressor that acts by binding to lysine demethylase 5B (KDM5B), Grainyhead-like 2 (GRHL2), B cell-specific moloney murine leukemia virus insertion site 1 (Bmi1), and suppressor of cytokine signaling 2 (SOCS2) (Bao et al., 2016; Das et al., 2017; Yu et al., 2017; Zhang et al., 2017). Our previous study demonstrated that FGF2 plays a protective role in IAV-induced lung injury; however, the underlying mechanisms regulating the FGF2 response remain unclear.

Here, we investigated the involvement of miR-194 in the process of FGF2 protection against IAV infection. We found that miR-194 was significantly downregulated during IAV infection. Moreover, miR-194 targeting of FGF2, a novel antiviral regulator, inhibited IAV replication by negatively regulating type I IFN production and RIG-I signaling. Importantly, inhibition of miR-194 alleviated IAV-induced ALI by promoting RIG-I-dependent antiviral pathways *in vitro* and *in vivo*.

## Materials and Methods

### miRNA Expression Profiling and Analysis

miRNA profiling was performed using GeneChip miRNA Array version 4.0 (Affymetrix, Santa Clara, CA, United States) under accession number GSE103009. The array comprised 30,434 mature miRNA sequences from the Sanger miRNA database (V.20). Microarray experiments were conducted according to the manufacturer’s instructions. miRNA probe outliers were defined as per the manufacturer’s instructions (Affymetrix) and further analyzed for data summarization, normalization, and quality control using the miRNA QC Tool software^1^. The significantly differentially expressed genes in the microarrays were analyzed (SAM, version 3.02) and selected using the threshold values of ≥2 and ≤ -2-fold change and a FDR significance level of <5%. The data were log2-transformed and median centered by genes using the Adjust Data function of CLUSTER 3.0 software, and then further analyzed by hierarchical clustering with average linkage. Finally, we performed tree visualizations using Java Tree View (Stanford University School of Medicine, Stanford, CA, United States).

### Viruses and Cells

The influenza virus used in this study was influenza A (H1N1) virus strain A/Beijing/501/2009 (BJ501). The strains were propagated in 9- to 11-day-old specific-pathogen-free (SPF) embryonated fowl eggs via the allantoic route. Virus titers were determined based on the 50% tissue infectious dose (TCID_50_) assay using Madin–Darby canine kidney (MDCK) cells according to the Reed–Muench method. MDCK, A549, and HEK-293T cells were purchased from the American Type Culture Collection (Manassas, VA, United States). MDCK and HEK-293T cells were cultured in DMEM (Gibco, Thermo Fisher Scientific, Grand Island, NY, United States), and A549 cells were cultured in DMEM/F-12 (1:1) basic (Gibco) supplemented with 10% fetal bovine serum (FBS) and 100 units penicillin-streptomycin/mL at 37°C in a humidified atmosphere of 5% (v/v) CO_2_.

### Mice

Four-week-old, wild-type (WT), SPF, C57BL/6 (abbreviated B6) female mice (Experimental Animal Center of the Academy of Military Medical Sciences, Beijing, China) were housed in the animal facility at the Beijing Institute of Microbiology and Epidemiology (Beijing, China) in accordance with institutional guidelines. All of the experimental protocols were approved by the Institutional Animal Care and Use Committees of the Beijing Institute of Microbiology and Epidemiology (ID: SYXK2015-008), and all of the experiments were performed in accordance with the approved guidelines.

### Mouse Infections

Four-week-old WT B6 mice were anesthetized with 50 μL 1% (w/v) pentobarbital sodium and then inoculated intranasally (i.n.) with H1N1 virus or mock-infected with allantoic fluid (AF) as a control. Survival rates, body weight changes, histology, and cytokine levels were evaluated as previously described (Mei et al., 2012; Wang et al., 2013). Mice receiving miR-194 agomir treatment or agomir negative control (4 nM; Guangzhou Ribobio, Guangzhou, China) were intravenously (i.v.) administered 12 h prior to, as well as 1 and 3 days after AF or virus (10^3^ TCID_50_ of A/Beijing/501/2009) instillation. In the rescue experiments, mice were administered miR-194 antagomir or antagomir negative control (8 nM, Guangzhou Ribobio) injected i.v. 12 h prior to, as well as 1 and 3 days after AF or virus (10^5^ TCID_50_ of A/Beijing/501/2009) instillation. For FGF2 reintroduction, WT B6 mice were inoculated i.v. with 4 nM miR-194 agonist plus 25 μg recombinant murine FGF2 protein (Cat. No. 450-33; PeproTech, Rocky Hill, NJ, United States) 12 h prior to, as well as 1 and 3 days after AF or virus (10^3^ TCID_50_ of A/Beijing/501/2009) instillation.

### Viral Titration

Virus titers were determined in supernatants of mouse lung homogenates from mice on day 5 after H1N1 infection, as previously described. Briefly, samples were added to the first column of wells on a 96-well plate incubated with MDCK cells, and then diluted 10-fold; infected cells were maintained in culture for 96 h. Virus titers were calculated using the Reed–Muench method and expressed as TCID_50_ per milliliter of supernatant.

### Survival Rate and Body Weight Changes

Four-week-old WT B6 mice were anesthetized with 50 μL 1% (w/v) pentobarbital sodium, and inoculated i.n. with virus or AF. The survival percentages and body weights in each group of 10 mice were monitored daily for 14 days. Survival data were analyzed by the Kaplan–Meier survival analysis using GraphPad Prism 5 software.

### Histological Examination

Four-week old B6 mice were treated i.n. with 10^3^ or 10^5^ TCID_50_ BJ501 H1N1 virus (or an equal volume of virus diluent) after being anesthetized with 50 μL 1% (w/v) pentobarbital sodium. The mice were sacrificed at 5 days post-infection (dpi), and the lungs were fixed in formalin and embedded in paraffin. Ultrathin sections were obtained and stained with hematoxylin and eosin. The inflammatory cells were counted and represented as cell numbers per 50× field.

### Antibodies and Reagents

The primary antibodies used in the analysis were Anti-RIG-I (D14G6), anti-tank binding kinase 1 (TBK1) (D1B4), anti-phospho-TBK1 (Ser172, D52C2), anti-IRF3 (D6I4C), anti-phospho-IRF3 (Ser396, 4D4G), anti-β-actin (13E5), and anti-rabbit IgG (Cell Signaling Technology, Danvers, MA, United States). The anti-FGF2 antibody (ab106425) was purchased from Abcam (Cambridge, United Kingdom). Western Chemiluminescent Horseradish Peroxidase Substrate was purchased from Millipore Corporation (Billerica, MA, United States).

### Western Blotting

All of the cells were lysed in RIPA buffer (Solarbio, Beijing, China) supplemented with protease and phosphatase inhibitor cocktail (100×; Thermo Fisher) and lysed for 10 min on ice. The supernatants were mixed with 1/4 volume of 5× loading dye. Then the mixtures were heated at 95°C and stored at -80°C. The samples were separated by sodium dodecyl sulfate-polyacrylamide gel electrophoresis and transferred onto a nitrocellulose filter membrane. The membranes were blocked with 5% non-fat milk (Becton Dickinson, Franklin Lakes, NJ, United States) in 1× Tris-buffered saline and 0.1% Triton 100 for 1 h while shaking at room temperature. The membranes were incubated with primary antibodies followed by horseradish peroxidase-conjugated secondary antibodies. The band was visualized using the Kodak film exposure detection system. The film was scanned and the band intensity was analyzed using Quantity One software.

### Quantitative Real-Time Polymerase Chain Reaction

Total RNA was obtained from cultured cells with TRIzol reagent (Invitrogen, Carlsbad, CA, United States). cDNA was generated by reverse transcription with commercial PrimeScript RT Master Mix (Takara, Tokyo, Japan). The miRNA and U6 primers were synthesized by Guangzhou Ribobio. The primer pairs for IFN-α, IFN-β, M1, FGF2, and GAPDH (Supplementary Table S1) were designed using Primer Premier Software 5.0 (Premier Biosoft International, Palo Alto, CA, United States) and synthesized by Invitrogen. The quantitative real-time PCR (qPCR) reaction was performed in triplicate wells of a 96-well reaction plate on an ABI 7500 PCR System (Applied Biosystems, Foster City, CA, United States). GAPDH was used as the endogenous control. The 2^-ΔΔCt^ method was used to calculate expression relative to the endogenous control. The quantification data were analyzed with ABI 7500 SDS software v.1.3.

### Luciferase Report Assay

WT or mutant of 3′ UTR sequences of FGF2 were ligated into the pmiR-RB-Reporter plasmid (Guangzhou Ribobio). To construct the mutant FGF2 3′ UTR, the sequences that interact with the seed sequence of miR-194 were mutated (from UGCUGUUAC to ACGACAAUG). HEK-293T cells were seeded in 96-well plates and cotransfected with 100 ng pmiR-RB-Reporter plasmid and 50 nM miR-194 agomir or negative control using riboFECT CP Transfection Kit (Guangzhou Ribobio). At 24 h post-transfection, the cells were harvested according to the manufacturer’s instructions (Promega, Madison, WI, United States), and firefly and Renilla luciferase activities were measured using a Dual-Luciferase Reporter Assay System (Promega) with the Promega GloMax 96 Microplate Luminometer (Promega). For the IFNB1 transcriptional activity assay, 100 ng pGL3-IFNB1 or control luciferase plasmids were cotransfected with 20 ng pRL-TK vector into the cells using jetPRIME Transfection Reagent (Polyplus, Illkirch, France). The luciferase and Renilla signals were measured using the Dual Luciferase Reporter Assay Kit (Promega) according to the protocol provided by the manufacturer.

### Cytokine and Chemokine Measurements in Cell Supernatant and Mouse Bronchoalveolar Lavage Fluid

A549 cells were seeded in 24-well plates and transfected with 50 nM miR-194 agomir or 100 nM inhibitor or negative control (Guangzhou Ribobio) using the riboFECT CP Transfection kit (Guangzhou Ribobio). At 24 h post-transfection, A549 cells were infected with BJ501 (MOI = 1), and cell culture supernatants were collected 24 h after BJ501 infection. The BALFs from 4-week-old WT B6 mice were collected 5 dpi. Samples were processed with the precoated human IFN-α ELISA kit (Dakewe, Shenzhen, China) and human IFN-β ELISA kit (Elabscience, Wuhan, Hubei, China).

### Statistical Analyses

All of the analyses were performed using GraphPad Prism 5.0 (GraphPad Software, San Diego, CA, United States). Survival data were analyzed using the Kaplan–Meier survival analysis. Differences were considered significant at *P* <0.05 with a two-tailed test. All of the experiments were performed in triplicate.

## Results

### miRNA Expression Altered during IAV Infection

**FIGURE 1:**
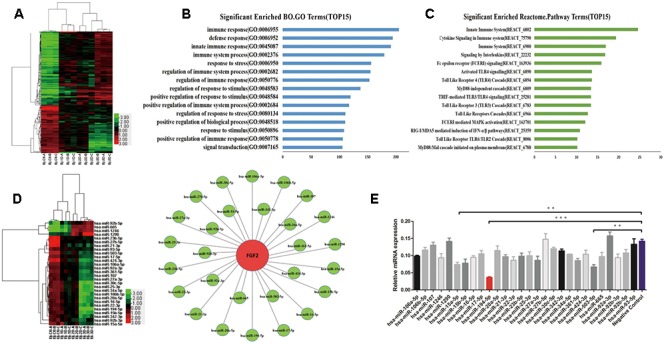
Influenza A virus (IAV) infection induces changes in miRNA expression. Microarray analysis for microRNAs (miRNAs) was performed with RNA extracts from influenza A virus (IAV)-infected A549 cells for 24, 48, and 72 h. The results are presented as the means ± standard deviations (SDs) of values obtained in three independent experiments. Statistical significance is indicated as ^∗^*P* <0.05, ^∗∗^*P* <0.01, and ^∗∗∗^*P* <0.001. **(A)** The cluster heatmap shows miRNAs with expression fold change >2 from microarray data (*P* <0.05). **(B)** Gene ontology analysis: Biological processes upregulated in IAV infection. **(C)** Enhancement score of pathways. KEGG pathways constructed from the differentially expressed miRNAs with scores listed as the top 15. **(D)** Generation of the IPA network highlighting the highly predicted fibroblast growth factor 2 (FGF2) target miRNAs (left) and the relative abundance of 28 miRNAs (right). **(E)** The results of the relative FGF2 expression levels in A549 cells overexpressing 28 miRNAs measured by quantitative real-time polymerase chain reaction (qPCR).

To investigate the roles of host miRNAs in IAV infection, miRNA microarray was performed using the Affymetrix GeneChip miRNA 4.0 array platform in human AECs (A549) uninfected or infected with H1N1, influenza virus strain A/Beijing/501/2009 (BJ501) for 24, 48, and 72 h. A total of 228 upregulated and 237 downregulated miRNAs in the infected cells were identified (fold change >2; *p* <0.05) and clustered (**Figure 1A**). To elucidate the roles of the differentially expressed miRNAs in response to influenza virus infection, potential targets of differentially expressed miRNAs were predicted using Targetscan 6.2 with a context score percentile >90. The predicted targets were obtained for the 465 differently expressed miRNAs and subjected to gene ontology (GO) and pathway analyses using the cut-off standard of *P* <0.01 and a false discovery rate (FDR) <1. The GO mapping revealed that the genes associated with the miRNA in the top network were functionally relevant to immune (GO 006955), defense (GO 006952), innate immune (GO 0045087), and stress responses (assist stress response) (GO 006950) (**Figure 1B**). Meanwhile, the identified miRNA-targeted genes were involved in 15 cellular innate response pathways including cytokine signaling, signaling by interleukins, TLR signaling, mitogen-activated protein kinase signaling, and RIG-I signaling (**Figure 1C**). We previously reported that administration of recombinant FGF2 protein markedly reduced mortality and the severity of lung injury in a preclinical model of IAV infection. Consistent with these previous results, FGF2 overexpression significantly decreased IAV replication (**Supplementary Figures S1A,B**). In addition, FGF2 enhanced IFNB1 transcriptional activity (**Supplementary Figure S1C**) and promoted type I IFN expression at the mRNA and protein levels (**Supplementary Figures S1D,E**). In this study, we investigated whether miRNAs could modulate FGF2 in IAV-induced ALI. Based on miRNA target analysis algorithms (miRanda, TargetScan and RNAhydrid), we obtained 28 differentially expressed miRNAs that were predicted to target FGF2 (**Figure 1D**). To validate that these miRNAs altered FGF2 expression, A549 cells were first transfected with miRNA mimic and then infected with BJ501. We found that miR-194 significantly downregulated FGF2 expression at 24 h post-infection (**Figure 1E**). These data indicate that miR-194 may play a pivotal role in regulating FGF2 expression.

### miR-194 Targets FGF2 in H1N1 Infection

**FIGURE 2:**
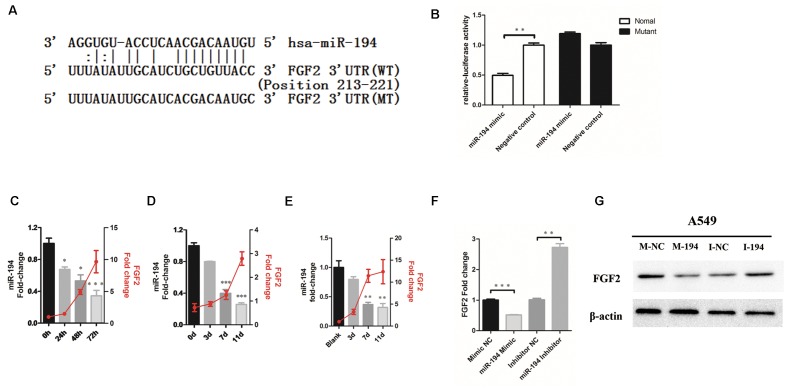
MicroRNA-194-5p targets FGF2 in H1N1 infection. **(A)** The predicted microRNA-194-5p (miR-194)-binding sequence in the 3′-untranslated region (UTR) of FGF2 mRNA is shown. Mutations were generated in the FGF2 3′-UTR sequence in the complementary sites for the seed regions in miR-194. **(B)** Analysis of luciferase activity. 293T cells were transfected with the WT-FGF2 3′-UTR or mutant-FGF2 3′-UTR with miR-194 mimic or negative control. ^∗^*P* <0.05. **(C,D)** The negative correlation between expression of miR-194 and FGF2 in infected A549 cells **(C)** and mouse lungs **(D)**. **(E)** The negative correlation between expression of miR-194 and FGF2 in infected mouse lung alveolar epithelial cells (AECs). **(F)** An inverse relationship between miR-194 and FGF2 was showed in A549 cells. **(G)** Western blot analysis of FGF2 protein expression in A549 cells.

To determine whether FGF2 is the functional target of miR-194 *in vitro*, target prediction was performed using the miRNA target analysis algorithms miRanda, TargetScan, and RNAhydrid. A common high-scoring candidate target was found on the 3′-UTR of FGF2 mRNA that was complementary to miR-194 (**Figure 2A**). A dual-luciferase reporter system revealed that miR-194 markedly repressed the reporter activity of the WT FGF2 3′-UTRs but not the mutant 3′-UTR (**Figure 2B**), indicating that FGF2 is a direct target of miR-194. We investigated the expression of FGF2 and miR-194 in A549 cells and mouse lung infected with IAV. The results showed that miR-194 was significantly downregulated in infected A549 cells and mouse lung, which corroborated the expression pattern observed using the microarray (**Figures 2C,D**). To determine the miR-194 expression level during IAV infection, we isolated AECs from the lungs of BJ501 virus-infected mice, and found that miR-194 expression was significantly decreased in AECs after BJ501 infection (**Figure 2E**). In addition, the expression of miR-194 negatively correlated with the FGF2 mRNA levels in infected A549 and mice (**Figures 2C–E**). Next, we explored the impact of altered miR-194 levels on FGF2 transcription and translation. The results showed that miR-194 overexpression markedly reduced the FGF2 expression at the mRNA and protein levels (**Figure 2F**). Furthermore, miR-194 inhibition abolished the functions (**Figure 2G**). These results suggest that FGF2 expression is regulated by miR-194.

### miR-194 Negatively Regulates IAV-Induced Type I IFNs in A549 Cells

**FIGURE 3:**
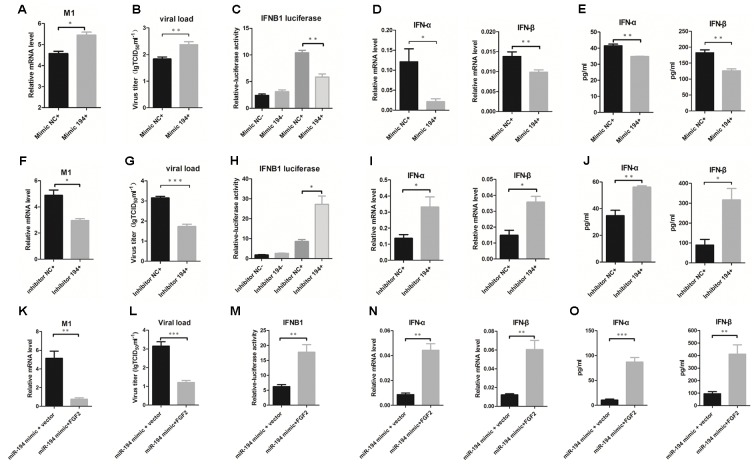
miR-194 negatively regulates IAV-induced type I interferons in A549 cells. Cells were transfected with miR-194 mimic or mimic negative control and miR-194 inhibitor or inhibitor negative control as indicated. For FGF2 reintroduction, PCDNA3. 1 (+)-FGF2 or vector were cotransfected with miR-194 mimic. After 24 h, cells were infected by BJ501 at a multiplicity of infection (MOI) = 1 for the indicated time. The results are presented as the means ± SDs of values obtained in three independent experiments. Statistical significance is indicated as follows: ^∗^*P* <0.05, ^∗∗^*P* <0.01, and ^∗∗∗^*P* <0.001. **(A,F,K)** The IAV titers in infected A549 cells as measured by qPCR. **(B,G,L)** The virus titers in infected A549, determined using the 50% tissue infectious dose (TCID_50_) assay. **(C,H,M)** Interferon B1 (IFNB1) transcriptional activity assay measured by dual luciferase reporter assay in HEK-293T cells. **(D,I,N)** IFN-α and IFN-β mRNA expression after 24 h post-infection determined by qPCR. **(E,J,O)** Levels of IFN-α and IFN-β cytokines in the cell culture supernatant, determined by enzyme-linked immunosorbent assay (ELISA).

To investigate the potential function of miR-194 during IAV infection, we further examined the effects of miR-194 on IAV replication in A549 cells. Overexpression of miR-194 in infected A549 cells facilitated influenza M1 expression (**Figure 3A**). Moreover, the TCID_50_ showed that miR-194 overexpression promoted IAV replication (**Figure 3B**). To gain insight into the mechanism underlying miR-194 modulation of viral replication, we analyzed IFNB1 transcriptional activity when miR-194 was overexpressed or inhibited. We found no significant difference in IFNB1 transcriptional activity in the uninfected control group. However, 24 h after IAV infection, overexpression of miR-194 suppressed IFNB1 transcriptional activity (**Figure 3C**). Consistent with the effect on IFNB1 transcriptional activity, miR-194 overexpression abrogated IFN-α and IFN-β mRNA expression in BJ501-infected A549 cells (**Figure 3D**). Similarly, the protein levels of IFN-α and IFN-β were significantly reduced in infected cells that overexpressed miR-194 (**Figure 3E**). Conversely, inhibition of miR-194 suppressed influenza M1 expression and virus replication (**Figures 3F,G**), and inhibition of miR-194 enhanced the activity of the IFNB1 promoter (**Figure 3H**). In addition, IFN-α and IFN-β mRNA or protein expression was significantly upregulated during inhibition of miR-194 (**Figures 3I,J**). Therefore, miR-194 inhibitor may attenuate IAV replication by enhancing the expression of type I IFNs. Reintroduction of FGF2 in miR-194-overexpressing cells abrogated the miR-194-induced effects on promoting IAV M1 expression, viral replication (**Figures 3K,L**), and suppression of IFNB1 transcriptional activity or production of type I IFNs (**Figures 3M–O**). Collectively, these results show that FGF2 reintroduction could abrogate the suppression of miR-194-induced type I IFNs, suggesting that FGF2 mediates the function of miR-194 in IAV infection.

### miR-194 Antagonism Suppresses the Pathogenesis of IAV Infection in Mouse Models

**FIGURE 4:**
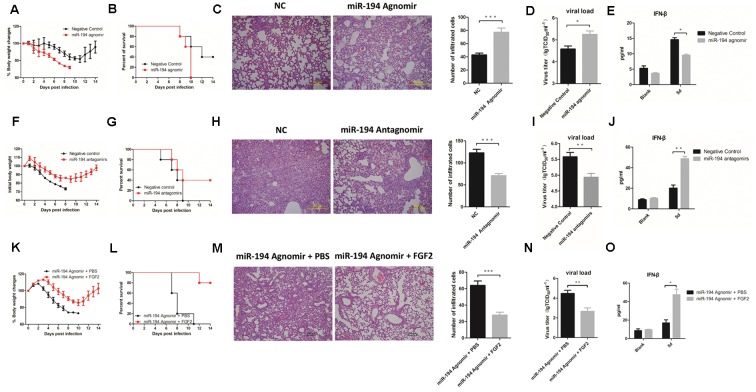
miR-194 antagonism alleviates lung injury induced by IAV. WT B6 mice were sequentially inoculated intravenously with 4 nM miR-194 agomir or negative control per mouse 12 h prior to, as well as 1 and 3 days after allantoic fluid (AF) or virus (10^3^ TCID_50_ of A/Beijing/501/2009) instillation. For miR-194 antagomir treatment, mice were sequentially inoculated intravenously with 8 nM miR-194 antagomir or negative control 12 h prior to, as well as 1 and 3 days after AF or virus (10^5^ TCID_50_ of A/Beijing/501/2009) instillation. For FGF2 administration, WT B6 mice were inoculated intravenously with 4 nM miR-194 agonism plus 25 μg recombinant murine FGF2 protein per mouse 12 h prior to, as well as 1 day and 3 days after AF or virus (10^3^ TCID_50_ of A/Beijing/501/2009) instillation. All of the data are shown as mean ± standard error of the mean (SEM), and independent experiments were repeated three times. ^∗^*P* <0.05, ^∗∗^*P* <0.01, and ^∗∗∗^*P* <0.001. **(A,F,K)** Weight changes in treated B6 mice (*n* = 5). **(B,G,L)** Survival rates of treated B6 mice (*n* = 5). **(C,H,M)** Hematoxylin and eosin (H&E) staining and quantification of lung tissues from treated mice at 5 days post-infection (dpi). **(D,I,N)** Virus titers of lung from treated mice (*n* = 6) determined based on the TCID_50_ assay at 5 dpi. **(E,J,O)** IFN-β cytokine levels in the bronchoalveolar lavage fluids (5 dpi) were determined by ELISA (*n* = 3).

To determine the regulatory role of miR-194 and its effects on disease parameters *in vivo*, we treated 4-week-old WT B6 mice with miR-194 agomir and antagomir. miR-194 expression was significantly elevated in miR-194 agomir-treated mice and was reduced in miR-194 antagomir-treated mice (**Supplementary Figure S2**). Interestingly, we found aggravated pathology in miR-194 agomir-treated mice compared to untreated mice, as determined by weight loss and survival rate (**Figures 4A,B**). Lung histopathology of miR-194 agomir-treated mice showed hyperemia of the alveolar wall and inflammatory infiltration (**Figure 4C**). In addition, we found a significant increase in virus titer and a significant decrease in IFN-β secretion in BALF (**Figures 4D,E**). By contrast, the groups pretreated or treated with miR-194 antagomir showed alleviation of body weight loss (**Figure 4F**) and had significantly higher survival rates following BJ501 infection (**Figure 4G**). Moreover, the negative control-pretreated mice showed greater lung pathology than the miR-194 antagomir-treated group (**Figure 4H**). Consistent with the results *in vitro*, miR-194 antagomir promoted IFN-β secretion in BALF and decreased virus titer in the lung (**Figures 4I,J**). These results suggest that miR-194 antagonism alleviates lung injury induced by BJ501. To observe the function of miR-194 and FGF2 *in vivo*, WT B6 mice were inoculated i.v. with 4 nM miR-194 agonism plus 25 μg FGF2 recombinant protein 12 h prior to, as well as 1 and 3 days after AF or virus (10^3^ TCID_50_ of A/Beijing/501/2009) instillation. Similar to the findings *in vitro*, FGF2 abrogated the miR-194 agonism that caused severe body weight loss, and the mice showed a better outcome of survival rates (**Figures 4K,L**). FGF2 administration improved the lung histopathology in infected mice, and the infiltrated leukocyte counts were significantly decreased (**Figure 4M**). Moreover, FGF2-administrated mice had a significantly decreased viral load, and the IFN-β secretion in BALF was significantly stimulated (**Figures 4N,O**). These results showed the involvement of FGF2 in the function of miR-194 *in vivo* following IAV infection.

### miR-194 Suppresses RIG-I Signaling

**FIGURE 5:**
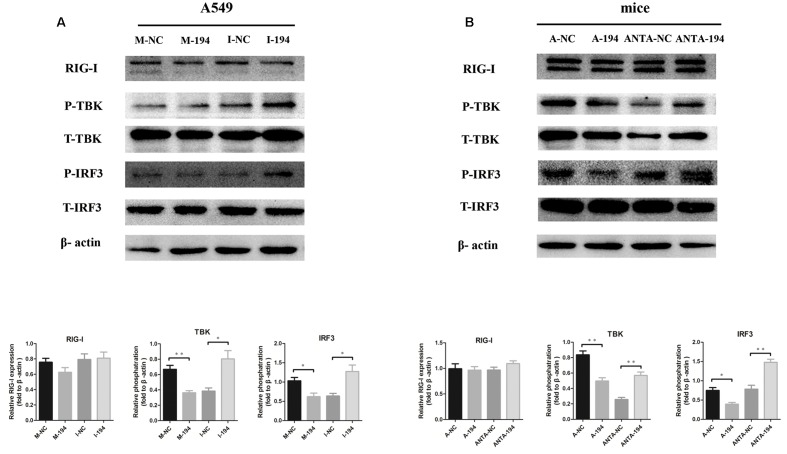
miR-194 suppresses retinoic acid inducible gene (RIG)-I signaling. Western blotting for RIG-I signaling pathway using β-actin as a loading control. Data represent three independent experiments. **(A)** A549 cells were transfected with miR-194 mimic (M-194) or mimic negative control (M-NC) and miR-194 inhibitor (I-194) or inhibitor negative control inhibitor (I-NC) as indicated. After 24 h, cells were infected by BJ501 at MOI = 1 for 24 h. **(B)** WT B6 mice were sequentially inoculated intravenously with 4 nM miR-194 agomir (A-194) or negative control (A-NC) and 8 nM miR-194 antagomir (ANTA-194) or negative control (ANTA-NC) 12 h prior to, as well as 1 and 3 days after AF or BJ501 instillation. Western blot analysis of signaling pathways in mice infected with 10^5^ TCID_50_ of BJ501 or mock infected at 5 dpi.

The RIG-I signaling pathway is crucial for viral production and for regulating the production of IFNs (Kato et al., 2005). We performed western blot analysis to investigate whether the RIG-I pathway participates in the miR-194-FGF2 axis with respect to the downstream signaling of type I IFNs. The results showed no significant difference in RIG-I expression when miR-194 was overexpressed or inhibited. Inhibition of miR-194 enhanced TBK1 and IRF3 phosphorylation, whereas miR-194 overexpression significantly suppressed TBK1 and IRF3 phosphorylation (**Figure 5A**). Similar results were obtained in miR-194 agomir-treated or antagomir-treated mice (**Figure 5B**). As expected, FGF2 overexpression activated RIG-I signaling (**Supplementary Figure S1F**). These results suggest that miR-194 inhibition promotes FGF2 expression, which enhances RIG-I signaling and affects production of type I IFNs.

## Discussion

We investigated the role of miR-194 during IAV infection. Inhibition of miR-194 enhanced type I IFN expression and type I IFN-mediated antiviral response, thereby suppressing viral replication both *in vitro* and *in vivo*. In addition, miR-194 negatively regulates IAV infection-triggered type I IFN production by suppressing the RIG-I pathway. Furthermore, miR-194 targeted FGF2 in IAV infection via a mechanism associated with RIG-I signaling.

FGF2, a pleiotropic signaling molecule, is involved in multiple biological processes including angiogenesis, embryonic development, and wound healing (Ortega et al., 1998; De Moerlooze et al., 2000; Nugent and Iozzo, 2000; Virag et al., 2007; Song et al., 2015). The cytosolic low-molecular-weight isoform FGF2 can stabilize RIG-I and prevent proteasome-mediated RIG-I degradation; furthermore, it serves as a negative regulator of the RIG-I-mediated signaling pathway, maintaining its autoinhibitory function (Liu et al., 2015). By contrast, other studies have revealed that administration of recombinant FGF2 protein significantly alleviated the severity of IAV-induced lung injury and promoted the survival of IAV-infected animals by mediating the recruitment of neutrophils. Here, our data revealed that overexpression of FGF2 (high-molecular-weight isoform) in A549 cells enhanced type I IFN production and suppressed virus replication in response to IAV challenge. We speculate that different molecular weight isoforms of FGF2 have different functions.

miRNAs represent a family of small non-coding RNAs that control the translation and transcription of various genes and have functions in different biological processes including the immune response, inflammation, and tumorigenesis (Bartel, 2004; Bushati and Cohen, 2007; Ebrahimi and Sadroddiny, 2015). miR-155 acts as a positive feedback regulator of type I IFN signaling in antiviral immunity by targeting SOCS1 (Wang et al., 2010). In addition, miR-146 simultaneously targets IL-1-associated kinase 1 and TRAF6 (Taganov et al., 2006), thereby suppressing pro-inflammation via TLR and RIG-I signaling. In addition, miR-15a/16 alters phagocytosis and bacterial clearance by targeting TLR4-associated pathways, affecting the survival of septic mice (Moon et al., 2014). Therefore, dozens of miRNAs with known functions in the immune system constitute promising drug targets for therapy.

miR-15/16 promotes tumor angiogenesis and metastasis by enhancing the expression of FGF2 (Xue et al., 2015), and miR-503 inhibits tumor angiogenesis and growth by simultaneously downregulating FGF2 expression (Zhou et al., 2013). Consistent with these results, our data showed that miR-15a and miR-503 could downregulate FGF2 expression. Moreover, we found that miR-194 significantly downregulated FGF2 expression following IAV infection. However, further investigation is required to understand the mechanisms underlying the downregulation of miR-194 in BJ501-infected mouse lung, and whether it involves endogenous antiviral activity or cell death. Using computational algorithms, we predicted that miR-194 targeted FGF2 directly and our experimental results confirmed that miR-194 significantly represses FGF2 expression by directly targeting FGF2. Inhibition of miR-194 enhanced type I IFN expression and type I IFN-mediated antiviral response, thus suppressing viral replication *in vitro* and *in vivo*. Furthermore, we showed that miR-194 antagonism alleviated IAV infection-induced lung injury, whereas miR-194 agonist administration aggravated the pathogenic disease. miR-194 targets many genes. For example, Zhang’s group found that miR-194 suppresses proliferation, migration, and invasion by targeting RAP2B in human bladder cancer (Zhang et al., 2016). In addition, miR-194 inhibited the epithelial-mesenchymal transition phenotype in gastric cancer cells by targeting FoxM1 (Li et al., 2014). Consistent with these reports, we showed that FGF2 is a novel target of miR-194, and such targeting affects the antiviral innate immunity in influenza H1N1 virus infection. Previous studies have shown that miR-194 inhibition might promote cell proliferation, migration, and the epithelial–mesenchymal transition process, a common feature during cancer progression. Our future studies will focus on the long-term effects of applying miR-194 antagomir to treat IAV infection *in vivo*. As miR-194 targets multiple genes, we speculate that these genes competitively bind miR-194, resulting in the incomplete suppression of a predominant functional gene, FGF2. Therefore, the fact that decreased miR-194 expression promotes virus clearance suggests that miR-194 antagonism might be a novel targeted approach for treating IAV infection. In addition, FGF2 reintroduction abrogated miR-194-induced type I IFN suppression and promoted virus clearance both *in vitro* and *in vivo*, suggesting that FGF2 mediates the function of miR-194 in IAV infection. Thus, miR-194 plays a crucial regulatory role in the FGF2-mediated antiviral response.

The presence of viral RNA in the cytosol initiates the innate immune response to the virus by activating three major intracellular immune pathways: RIG-I proteins, TLRs (primarily TLR3 and TLR7), and inflammasomes (Iwasaki and Pillai, 2014). RIG-I, an RNA helicase, acts as a critical mediator of the host response to RNA viruses, including IAV, through its ability to recognize 5’ capped single-stranded RNA in the infected cell cytoplasm (Pichlmair et al., 2006). Binding of viral RNA to helicase domains on RIG-I triggers its interaction with MAVS, which leads to the translocation of IRF3/7 to the nucleus and generation of type I and III IFNs (IFN-α/β and IFN-λ) (Durbin et al., 2013). In this study, we revealed that miR-194 negatively regulates IAV infection-triggered type I IFN production by suppressing the RIG-I pathway. FGF2 blocks miR-194 induced type I IFN decreases while activate RIG-I signaling, indicating that miR-194 is directly mediated by the target FGF2. Our findings suggest that activation of the RIG-I signaling pathway by FGF2 may have a pivotal role in the miR-194-mediated change in type I IFN production in IAV infection.

To the best of our knowledge, this report is the first to identify FGF2 as a novel target of miR-194, and to show that inhibition of miR-194 alleviates IAV-induced ALI by promoting RIG-I-dependent antiviral pathways. This study broadens our understanding of the roles of miR-194 in the interaction between the host and virus.

## Author Contributions

KW, CL, HG, LZ, and MX contributed to the experiments. KW and CL analyzed the data. KW wrote the manuscript. KW contributed reagents/materials/analysis tools. PY and XW designed the experiments and extensively reviewed/edited the manuscript.

## Conflict of Interest Statement

The authors declare that the research was conducted in the absence of any commercial or financial relationships that could be construed as a potential conflict of interest.

## Footnotes

1 www.affymetrix.com

## Supplementary Material

FIGURE S1 FGF2 suppresses IAV replication by enhancing type I IFN RIG-I signaling. A549 cells were transfected with PCDNA3. 1 (+)-vector or PCDNA3. 1 (+)-FGF2 as indicated. After 14 h, cells were infected by BJ501 at MOI = 1 for the indicated time. **(A)** IAV titers in infected A549 cells as measured by qPCR. **(B)** The virus titers in infected A549 cells determined using the TCID_50_ assay. **(C)** IFNB1 transcriptional activity assay measured by dual luciferase reporter assay in HEK-293T cells. **(D)** IFN-α and IFN-β mRNA expression after 24 h post-infection determined by qPCR. **(E)** Levels of IFN-α and IFN-β cytokines in the cell culture supernatant, as determined by ELISA. **(F)** Western blotting for RIG-I signaling pathway using β-actin as the loading control. Data represent three independent experiments.

FIGURE S2 miR-194 expression after agomir or antagomir treatment *in vivo.* WT B6 mice were sequentially inoculated intravenously with 4 nM miR-194 agomir or negative control 12 h prior to, as well as 1 and 3 days after AF or virus (10^3^ TCID_50_ of A/Beijing/501/2009) instillation. While for miR-194 antagnomir, mice were sequentially inoculated intravenously with 8 nM miR-194 antagomir or negative control 12 h prior to, as well as 1 and 3 days after AF or virus (10^5^ TCID_50_ of A/Beijing/501/2009) instillation. All data are shown as mean ± SEM, and independent experiments were repeated three times. ^∗^*P* <0.05, ^∗∗^*P* <0.01, and ^∗∗∗^*P* <0.001. **(A)** The expression of miR-194 in infected miR-194 agomir-treated mouse lung at 5 dpi (*n* = 5). **(B)** The expression of miR-194 in infected miR-194 antagomir-treated mouse lung at 5 dpi (*n* = 5).

## References

[B1] Bao J. Zou J. H. Li C. Y. Zheng G. Q. (2016). miR-194 inhibits gastric cancer cell proliferation and tumorigenesis by targeting KDM5B. *Eur. Rev. Med. Pharmacol. Sci.* 20 4487–4493. 27874950

[B2] Bartel D. P. (2004). MicroRNAs: genomics, biogenesis, mechanism, and function. *Cell* 116 281–297. 10.1016/S0092-8674(04)00045-5 14744438

[B3] Bushati N. Cohen S. M. (2007). microRNA functions. *Annu. Rev. Cell Dev. Biol.* 23 175–205. 10.1146/annurev.cellbio.23.090506.123406 17506695

[B4] Cao B. Li X. W. Mao Y. Wang J. Lu H. Z. Chen Y. S. (2009). Clinical features of the initial cases of 2009 pandemic influenza A (H1N1) virus infection in China. *N. Engl. J. Med.* 361 2507–2517. 10.1056/NEJMoa0906612 20007555

[B5] Das R. Gregory P. A. Fernandes R. C. Denis I. Wang Q. Townley S. L. (2017). MicroRNA-194 promotes prostate cancer metastasis by inhibiting SOCS2. *Cancer Res.* 77 1021–1034. 10.1158/0008-5472.CAN-16-2529 28011622

[B6] De Moerlooze L. Spencer-Dene B. Revest J. M. Hajihosseini M. Rosewell I. Dickson C. (2000). An important role for the IIIb isoform of fibroblast growth factor receptor 2 (FGFR2) in mesenchymal-epithelial signalling during mouse organogenesis. *Development* 127 483–492. 10631169 10.1242/dev.127.3.483

[B7] Durbin R. K. Kotenko S. V. Durbin J. E. (2013). Interferon induction and function at the mucosal surface. *Immunol. Rev.* 255 25–39. 10.1111/imr.12101 23947345 PMC5972370

[B8] Ebrahimi A. Sadroddiny E. (2015). MicroRNAs in lung diseases: recent findings and their pathophysiological implications. *Pulm. Pharmacol. Ther.* 34 55–63. 10.1016/j.pupt.2015.08.007 26319446

[B9] Globinska A. Pawelczyk M. Kowalski M. L. (2014). MicroRNAs and the immune response to respiratory virus infections. *Exp. Rev. Clin. Immunol.* 10 963–971. 10.1586/1744666X.2014.913482 24784476

[B10] Herold S. Becker C. Ridge K. M. Budinger G. R. (2015). Influenza virus-induced lung injury: pathogenesis and implications for treatment. *Eur. Respir. J.* 45 1463–1478. 10.1183/09031936.00186214 25792631

[B11] Iwasaki A. Pillai P. S. (2014). Innate immunity to influenza virus infection. *Nat. Rev. Immunol.* 14 315–328. 10.1038/nri3665 24762827 PMC4104278

[B12] Jain S. Kamimoto L. Bramley A. M. Schmitz A. M. Benoit S. R. Louie J. (2009). Hospitalized patients with 2009 H1N1 influenza in the United States, April-June 2009. *N. Engl. J. Med.* 361 1935–1944. 10.1056/NEJMoa0906695 19815859

[B13] Kato H. Sato S. Yoneyama M. Yamamoto M. Uematsu S. Matsui K. (2005). Cell type-specific involvement of RIG-I in antiviral response. *Immunity* 23 19–28. 10.1016/j.immuni.2005.04.010 16039576

[B14] Li Z. Ying X. Chen H. Ye P. Shen Y. Pan W. (2014). MicroRNA-194 inhibits the epithelial-mesenchymal transition in gastric cancer cells by targeting FoxM1. *Dig. Dis. Sci.* 59 2145–2152. 10.1007/s10620-014-3159-6 24748184

[B15] Liu X. Luo D. Yang N. (2015). Cytosolic low molecular weight FGF2 orchestrates RIG-I-mediated innate immune response. *J. Immunol.* 195 4943–4952. 10.4049/jimmunol.1501503 26466960 PMC4637180

[B16] Loveday E. K. Svinti V. Diederich S. Pasick J. Jean F. (2012). Temporal- and strain-specific host microRNA molecular signatures associated with swine-origin H1N1 and avian-origin H7N7 influenza A virus infection. *J. Virol.* 86 6109–6122. 10.1128/JVI.06892-11 22438559 PMC3372180

[B17] Mei J. Liu Y. Dai N. Hoffmann C. Hudock K. M. Zhang P. (2012). Cxcr2 and Cxcl5 regulate the IL-17/G-CSF axis and neutrophil homeostasis in mice. *J. Clin. Investig.* 122 974–986. 10.1172/JCI60588 22326959 PMC3287232

[B18] Moon H. G. Yang J. Zheng Y. Jin Y. (2014). miR-15a/16 regulates macrophage phagocytosis after bacterial infection. *J. Immunol.* 193 4558–4567. 10.4049/jimmunol.1401372 25261473 PMC4216178

[B19] Nugent M. A. Iozzo R. V. (2000). Fibroblast growth factor-2. *Int. J. Biochem. Cell Biol.* 32 115–120. 10.1016/S1357-2725(99)00123-5 10687947

[B20] Ortega S. Ittmann M. Tsang S. H. Ehrlich M. Basilico C. (1998). Neuronal defects and delayed wound healing in mice lacking fibroblast growth factor 2. *Proc. Natl. Acad. Sci. U.S.A.* 95 5672–5677. 10.1073/pnas.95.10.5672 9576942 PMC20437

[B21] Pichlmair A. Schulz O. Tan C. P. Naslund T. I. Liljestrom P. Weber F. (2006). RIG-I-mediated antiviral responses to single-stranded RNA bearing 5′-phosphates. *Science* 314 997–1001. 10.1126/science.1132998 17038589

[B22] Rehwinkel J. Tan C. P. Goubau D. Schulz O. Pichlmair A. Bier K. (2010). RIG-I detects viral genomic RNA during negative-strand RNA virus infection. *Cell* 140 397–408. 10.1016/j.cell.2010.01.020 20144762

[B23] Rosenberger C. M. Podyminogin R. L. Diercks A. H. Treuting P. M. Peschon J. J. Rodriguez D. (2017). miR-144 attenuates the host response to influenza virus by targeting the TRAF6-IRF7 signaling axis. *PLOS Pathog.* 13:e1006305. 10.1371/journal.ppat.1006305 28380049 PMC5393898

[B24] Short K. R. Kroeze E. J. Fouchier R. A. Kuiken T. (2014). Pathogenesis of influenza-induced acute respiratory distress syndrome. *Lancet Infect. Dis.* 14 57–69. 10.1016/S1473-3099(13)70286-X 24239327

[B25] Song X. Dai D. He X. Zhu S. Yao Y. Gao H. (2015). Growth factor FGF2 cooperates with interleukin-17 to repair intestinal epithelial damage. *Immunity* 43 488–501. 10.1016/j.immuni.2015.06.024 26320657

[B26] Taganov K. D. Boldin M. P. Chang K. J. Baltimore D. (2006). NF-kappaB-dependent induction of microRNA miR-146, an inhibitor targeted to signaling proteins of innate immune responses. *Proc. Natl. Acad. Sci. U.S.A.* 103 12481–12486. 10.1073/pnas.0605298103 16885212 PMC1567904

[B27] Tavares L. P. Teixeira M. M. Garcia C. C. (2017). The inflammatory response triggered by Influenza virus: a two edged sword. *Inflam. Res.* 66 283–302. 10.1007/s00011-016-0996-0 27744631

[B28] Virag J. A. Rolle M. L. Reece J. Hardouin S. Feigl E. O. Murry C. E. (2007). Fibroblast growth factor-2 regulates myocardial infarct repair: effects on cell proliferation, scar contraction, and ventricular function. *Am. J. Pathol.* 171 1431–1440. 10.2353/ajpath.2007.070003 17872976 PMC2043505

[B29] Wang P. Hou J. Lin L. Wang C. Liu X. Li D. (2010). Inducible microRNA-155 feedback promotes type I IFN signaling in antiviral innate immunity by targeting suppressor of cytokine signaling 1. *J. Immunol.* 185 6226–6233. 10.4049/jimmunol.1000491 20937844

[B30] Wang W. Yang P. Zhong Y. Zhao Z. Xing L. Zhao Y. (2013). Monoclonal antibody against CXCL-10/IP-10 ameliorates influenza A (H1N1) virus induced acute lung injury. *Cell Res.* 23 577–580. 10.1038/cr.2013.25 23419516 PMC3616436

[B31] Weber-Gerlach M. Weber F. (2016). Standing on three legs: antiviral activities of RIG-I against influenza viruses. *Curr. Opin. Immunol.* 42 71–75. 10.1016/j.coi.2016.05.016 27318973

[B32] Wolf S. Wu W. Jones C. Perwitasari O. Mahalingam S. Tripp R. A. (2016). MicroRNA regulation of human genes essential for influenza A (H7N9) replication. *PLOS ONE* 11:e0155104. 10.1371/journal.pone.0155104 27166678 PMC4864377

[B33] Xue G. Yan H. L. Zhang Y. Hao L. Q. Zhu X. T. Mei Q. (2015). c-Myc-mediated repression of miR-15-16 in hypoxia is induced by increased HIF-2alpha and promotes tumor angiogenesis and metastasis by upregulating FGF2. *Oncogene* 34 1393–1406. 10.1038/onc.2014.82 24704828

[B34] Yu X. An J. Hua Y. Li Z. Yan N. Fan W. (2017). MicroRNA-194 regulates keratinocyte proliferation and differentiation by targeting Grainyhead-like 2 in psoriasis. *Pathol. Res. Pract.* 213 89–97. 10.1016/j.prp.2016.11.020 28040329

[B35] Zhang M. Zhuang Q. Cui L. (2016). MiR-194 inhibits cell proliferation and invasion via repression of RAP2B in bladder cancer. *Biomed. Pharmacother.* 80 268–275. 10.1016/j.biopha.2016.03.026 27133066

[B36] Zhang X. Wei C. Li J. Liu J. Qu J. (2017). MicroRNA-194 represses glioma cell epithelial-to-mesenchymal transition by targeting Bmi1. *Oncol. Rep.* 37 1593–1600. 10.3892/or.2017.5376 28098896

[B37] Zhou B. Ma R. Si W. Li S. Xu Y. Tu X. (2013). MicroRNA-503 targets FGF2 and VEGFA and inhibits tumor angiogenesis and growth. *Cancer Lett.* 333 159–169. 10.1016/j.canlet.2013.01.028 23352645

