# Supplementary figures and images for: miR-194 Inhibits Innate Antiviral Immunity by Targeting FGF2 in Influenza H1N1 Virus Infection
Source: Front Microbiol. 2017 Nov 7;8:2187. doi: 10.3389/fmicb.2017.02187 (PMC5674008; doi:10.3389/fmicb.2017.02187)

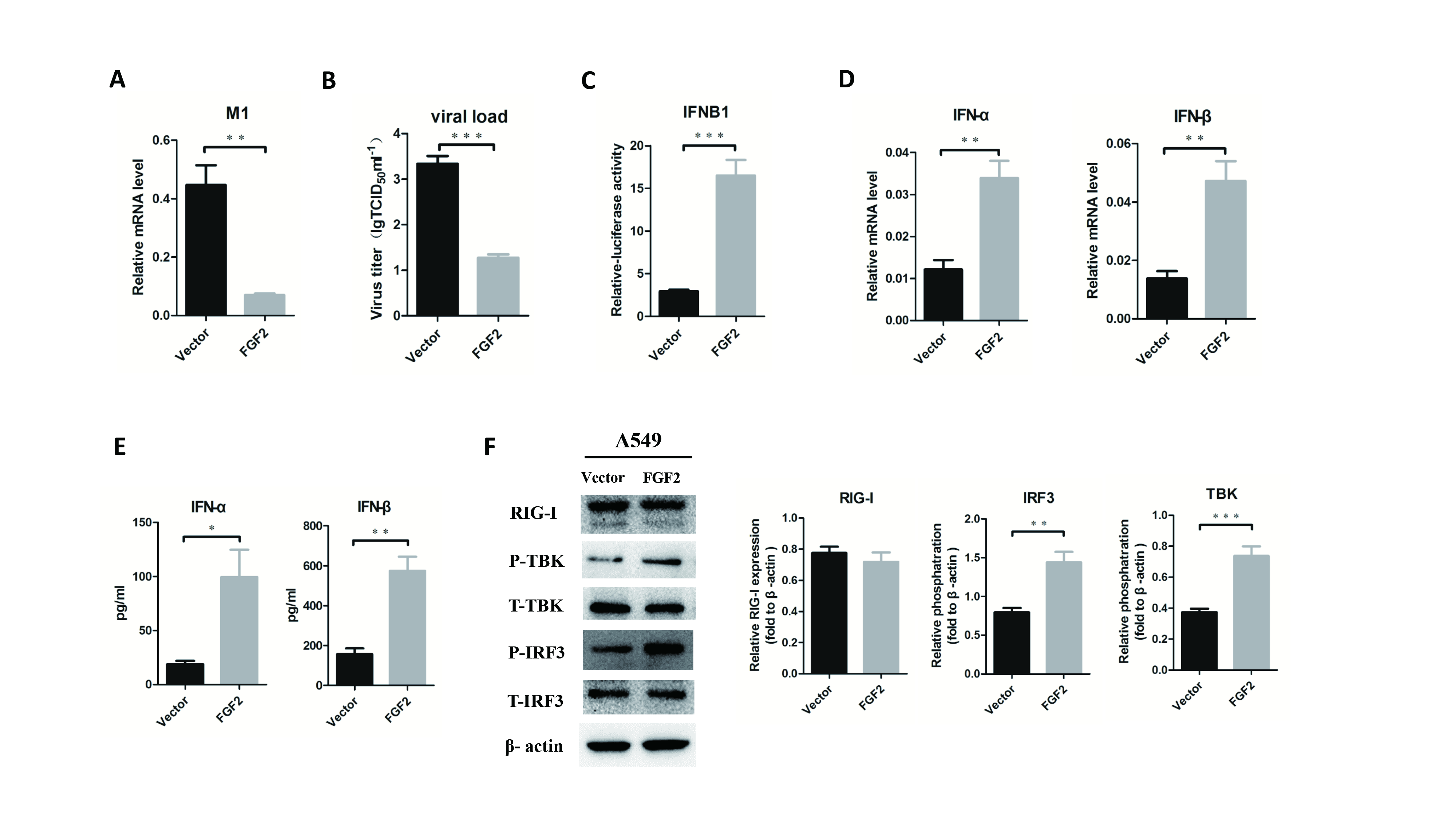

Supplement: FIGURE S1 — FGF2 suppresses IAV replication by enhancing type I IFN RIG-I signaling. A549 cells were transfected with PCDNA3. 1 (+)-vector or PCDNA3. 1 (+)-FGF2 as indicated. After 14 h, cells were infected by BJ501 at MOI = 1 for the indicated time. (A) IAV titers in infected A549 cells as measured by qPCR. (B) The virus titers in infected A549 cells determined using the TCID50 assay. (C) IFNB1 transcriptional activity assay measured by dual luciferase reporter assay in HEK-293T cells. (D) IFN-α and IFN-β mRNA expression after 24 h post-infection determined by qPCR. (E) Levels of IFN-α and IFN-β cytokines in the cell culture supernatant, as determined by ELISA. (F) Western blotting for RIG-I signaling pathway using β-actin as the loading control. Data represent three independent experiments. [file Image_1.TIF]

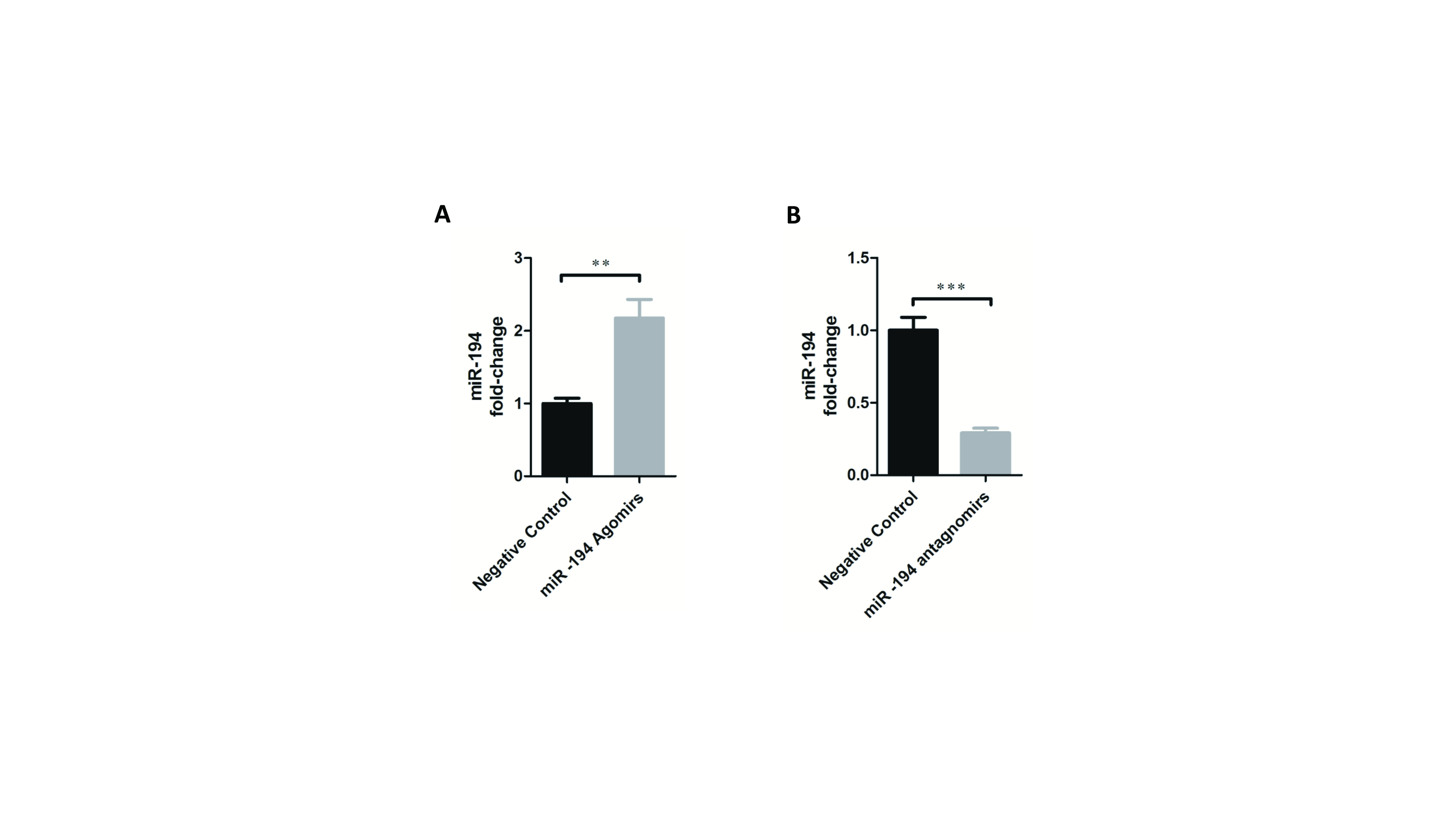

Supplement: FIGURE S2 — miR-194 expression after agomir or antagomir treatment in vivo. WT B6 mice were sequentially inoculated intravenously with 4 nM miR-194 agomir or negative control 12 h prior to, as well as 1 and 3 days after AF or virus (103 TCID50 of A/Beijing/501/2009) instillation. While for miR-194 antagnomir, mice were sequentially inoculated intravenously with 8 nM miR-194 antagomir or negative control 12 h prior to, as well as 1 and 3 days after AF or virus (105 TCID50 of A/Beijing/501/2009) instillation. All data are shown as mean ± SEM, and independent experiments were repeated three times. ∗P <0.05, ∗∗P <0.01, and ∗∗∗P <0.001. (A) The expression of miR-194 in infected miR-194 agomir-treated mouse lung at 5 dpi (n = 5). (B) The expression of miR-194 in infected miR-194 antagomir-treated mouse lung at 5 dpi (n = 5). [file Image_2.TIF]
