# Supplementary material for: miR-194 Inhibits Innate Antiviral Immunity by Targeting FGF2 in Influenza H1N1 Virus Infection
Source: Front Microbiol. 2017 Nov 7;8:2187. doi: 10.3389/fmicb.2017.02187 (PMC5674008; doi:10.3389/fmicb.2017.02187)
Supplement: Supplementary file 3 [file Table_1.DOCX]

**Supplemental Tables**

**Table S1.Primer pairs used for real-time PCR**

| Gene | Forward primer | Reverse primer |
| --- | --- | --- |
| Mouse FGF2 | 5’-GTGTGTGCTAACCGTTACCT-3’ | 5’-GTGTGTGCTAACCGTAACCT-3’ |
| Mouse GAPDH | 5’-ACAGCCGCATCTTCTTGTGCAGTG-3’ | 5’-GGCCTTGACTGTGCCGTTACCT-3’ |
| Mouse IFN-α | 5’-GCAACCCTCCTAGACTCATTCT-3’ | 5’-CCAGCAGGGCGTCTTCCT-3’ |
| Mouse IFN-β | 5’-CGGACTTCAAGATCCCTATGGA-3’ | 5’-TGGCAAAGGCAGTGTAACTCTTC-3’ |
| Human FGF2 | 5’-GGCCGGGGCCGGGGCCGTGC-3’ | 5’-GCTGTACTGCAAAAACGGGG-3’ |
| Human IFN-α | 5’-GTGAGGAAATACTTCCAAAGA-3’ | 5’-TCTCATGATTTCTGCTCTGACA-3’ |
| Human IFN-β | 5’-AGCTGAAGCAGTTCCAGAAG-3’ | 5’-AGTCTCATTCCAGCCAGTGC-3’ |
| M1 | 5’-AAGACCAATCCTGTCACCTCTG-3’ | 5’-CAAAACGTCTACGCTGCAGTCC-3’ |
| Human GAPDH | 5’-GGTGGTCTCCTCTGACTTCAACA-3’ | 5’-GTTGCTGTAGCCAAATTCGTTGT-3’ |
